# CMV seropositivity and T‐cell senescence predict increased cardiovascular mortality in octogenarians: results from the Newcastle 85+ study

**DOI:** 10.1111/acel.12430

**Published:** 2015-12-22

**Authors:** Ioakim Spyridopoulos, Carmen Martin‐Ruiz, Catharien Hilkens, Mohammad E. Yadegarfar, John Isaacs, Carol Jagger, Tom Kirkwood, Thomas von Zglinicki

**Affiliations:** ^1^ Newcastle University Institute for AgeingNewcastle UniversityNewcastle upon TyneNE4 5PLUK; ^2^ Institute of Genetic MedicineNewcastle UniversityNewcastle upon TyneNE4 5PLUK; ^3^ Institute of NeuroscienceNewcastle UniversityNewcastle upon TyneNE4 5PLUK; ^4^ Institute of Cellular MedicineNewcastle UniversityNewcastle upon TyneNE4 5PLUK; ^5^ Institute of Health and SocietyNewcastle UniversityNewcastle upon TyneNE4 5PLUK; ^6^ Institute for Cell and Molecular BiosciencesNewcastle UniversityNewcastle upon TyneNE4 5PLUK

**Keywords:** aging, CD4, CD8, coronary heart disease, cytomegalovirus, immunosenescence, octogenarians, survival, T lymphocytes

## Abstract

Although chronic infection with cytomegalovirus (CMV) is known to drive T lymphocytes toward a senescent phenotype, it remains controversial whether and how CMV can cause coronary heart disease (CHD). To explore whether CMV seropositivity or T‐cell populations associated with immunosenescence were informative for adverse cardiovascular outcome in the very old, we prospectively analyzed peripheral blood samples from 751 octogenarians (38% males) from the Newcastle 85+ study for their power to predict survival during a 65‐month follow‐up (47.3% survival rate). CMV‐seropositive participants showed a higher prevalence of CHD (37.7% vs. 26.7%, *P* = 0.030) compared to CMV‐seronegative participants together with lower CD4/CD8 ratio (1.7 vs. 4.1, *P* < 0.0001) and higher frequencies of senescence‐like CD4 memory cells (41.1% vs. 4.5%, *P* < 0.001) and senescence‐like CD8 memory cells (TEMRA, 28.1% vs. 6.7%, *P* < 0.001). CMV seropositivity was also associated with increased six‐year cardiovascular mortality (HR 1.75 [1.09–2.82], *P* = 0.021) or death from myocardial infarction and stroke (HR 1.89 [107–3.36], *P* = 0.029). Gender‐adjusted multivariate Cox regression analysis revealed that low percentages of senescence‐like CD4 T cells (HR 0.48 [0.32–0.72], *P* < 0.001) and near‐senescent (CD27 negative) CD8 T cells (HR 0.60 [0.41–0.88], *P* = 0.029) reduced the risk of cardiovascular death. For senescence‐like CD4, but not near‐senescent CD8 T cells, these associations remained robust after additional adjustment for CMV status, comorbidities, and inflammation markers. We conclude that CMV seropositivity is linked to a higher incidence of CHD in octogenarians and that senescence in both the CD4 and CD8 T‐cell compartments is a predictor of overall cardiovascular mortality as well as death from myocardial infarction and stroke.

## Introduction

Human cytomegalovirus (CMV) is a ubiquitous herpes virus and shares a high prevalence in developed countries (Crough & Khanna, 2009). A growing body of evidence suggests an important role of CMV during aging (Pawelec *et al*., 2009; Solana *et al*., 2012). Seropositivity for CMV is one of the parameters in the immune risk profile (IRP), associated with increased mortality in longitudinal studies in octo‐ and nonagenarians (Nilsson *et al*., 2003). While the IRP was present in only 20% of the 85‐year‐olds in the Swedish OCTO/NONA series, CMV seropositivity is present in approx. 80–90% of octogenarians (Olsson *et al*., 2000; Wikby *et al*., 2002; Nilsson *et al*., 2003). Phenotypical changes in the immune system attributable to CMV are most visible in the T‐cell compartment, coinciding with clonal expansion and preferential expansion of CD8^+^CD45^RA+^CD27^−^ cytolytic T cells (Khan *et al*., 2002; Kuijpers *et al*., 2003). CD8 T‐cell responses in CMV‐seropositive elderly are characterized by an accumulation of dysfunctional T cells with short telomeres and low proliferation potential, often considered as replicative senescent (Ouyang *et al*., 2003; Griffiths *et al*., 2013). Clinically, CMV has been linked to an increased incidence of coronary heart disease (CHD) in a number of studies (Ridker *et al*., 1998; Muhlestein *et al*., 2000; Sorlie *et al*., 2000; Blankenberg *et al*., 2001; Simanek *et al*., 2009). It has been proposed that CMV‐driven cardiovascular mortality might be the main cause for the observed increase in mortality in CMV‐seropositive people over the age of 65 years (Savva *et al*., 2013). We have shown previously that in CMV‐seropositive patients with CHD, the degree of telomere erosion in CD8 cells correlates with deterioration of left ventricular function, linking immunosenescence and cardiovascular disease (Spyridopoulos *et al*., 2009; Hoffmann *et al*., 2015). In offspring of longevity families, individuals do not show CMV‐associated changes to their immune signatures, suggesting a genetic component in the immune response to CMV (Derhovanessian *et al*., 2010). The goal of our study was to evaluate whether in octogenarians CMV seropositivity and T‐cell senescence are independent predictors of all‐cause and especially cardiovascular and CHD‐mediated mortality.

We prospectively analyzed peripheral blood samples from 749 octogenarians aged 85 years, approx. 38.5% were male (Table 1). A total of 85.6% of participants were seropositive for cytomegalovirus (CMV). There were no differences between CMV‐positive and CMV‐negative groups regarding gender, cardiovascular risk factors, and prevalence of a wide range of age‐related diseases, with the sole exception of a higher prevalence of ischemic heart disease in CMV‐positive study participants (37.7% vs. 26.7%, *P* = 0.030, Table 1). CMV seropositivity was associated with major changes in the T‐cell repertoire including a decreased CD4/CD8 cell ratio and increased frequencies of CD4 and CD8 effector memory (TEMRA) cells showing a senescence‐like phenotype characterized here by loss of CD27 and CD28 expression in the CD4 subset and by loss of CD45RO and CD27 in CD8 T cells. These surface marker combinations have been associated with low proliferative potential, short telomeres, low telomerase activity, and high expression of KLRG1, an inhibitor of AKT‐mediated proliferation (Henson & Akbar, 2009), and are thus regarded as good markers of a senescence‐like T‐cell phenotype. However, there were no differences in the levels of inflammatory markers at basal levels or following leukocyte stimulation by LPS (Table S1). Males and females differed in their CD4/CD8 ratios, but not in the frequencies of senescent CD4 or CD8 cells or in the levels of inflammation markers (Table S2).

**Table 1:** Patient characteristics (*n *= 749)

Parameter	CMV^−^ (*n *= 108)	CMV^+^ (*n *= 641)	*P*
Male gender (%)	42.6	37.8	0.339^a^
BMI (median, IQR)	23.93 (21.65–26.21)	24.36 (21.58–27.33)	0.170^b^
Smokers			
Never (%)	32.4	36.2	0.902^a^
Current (%)	5.6	5.5	
Former – Regular (%)	56.5	53.0	
Former – Occasional (%)	5.6	5.3	
Institutional housing (%)	4.6	8.9	0.137^a^
Diabetes (%)	14.8	13.4	0.695^a^
Hypertension (%)	51.9	58.0	0.230^a^
Total Cholesterol (median, IQR)	4.70 (4.00–5.60)	4.80 (4.00–5.70)	0.880^b^
Cardiovascular disease (%)	51.4	54.0	0.614^a^
Any atherosclerotic disease (%)	44.4	49.0	0.382^a^
Coronary heart disease (%)	26.7	37.7	**0.030** a
Cerebrovascular disease (%)	23.1	19.8	0.425^a^
Peripheral vascular disease (%)	6.5	6.9	0.884^a^
Heart failure (%)	10.2	11.4	0.714^a^
Rheumatoid arthritis (%)	2.8	3.0	0.916^a^
Cancer, any (%)	27.8	23.9	0.382^a^
Cancer, any (Excluding B&S, <5 years since diagnosis) (%)	9.3	6.1	0.217^a^
Anemia (WHO's Guideline) (%)	34.7	31.2	0.548^a^
Renal Impairment ‐ CKD‐Epi (%)	23.1	24.3	0.793^a^
Follow‐up (months, median (IQR))	65.07 (43.06–71.38)	65.02 (34.04–71.28)	0.491^b^
Survival (%)	49.1	47	0.684^a^

a Chi‐square.

b Mann–Whitney U‐test.

*P* values <0.05 are indicated in bold.

We assessed the impact of CMV status and T‐cell subset fractions on all‐cause mortality in octogenarians from the Newcastle 85+ study for which complete data for all leukocyte parameters, CMV status, and confounding factors (comorbidity, basal interleukin‐6 and TNF‐α) were available (*n *= 594). In gender‐adjusted log‐rank analyses, neither CMV status (*P* = 0.235) nor CD4/CD8 ratio (*P* = 0.184) was associated with all‐cause mortality, but frequencies of senescence‐like CD4 memory cells (CD4^+^CD45^RO+^CD27^−^CD28^−^) were weakly predictive of survival (*P* = 0.042). Similarly, a Cox regression model with leukocyte subsets entered singly and adjusting for gender revealed only a low percentage of senescence‐like CD4 memory cells to be significant predictors of long‐term mortality (HR 0.69 [0.52–0.92], *P* = 0.012), but this disappeared after adjustment for CMV status (Table S3). We conclude that in our octogenarian cohort, the impact of CMV on all‐cause mortality was low and mediated by CD4 T‐cell senescence.

To address the association between CMV infection, T‐cell senescence, and cardiovascular morbidity and mortality, we first compared T‐cell parameters and inflammation markers between CMV‐seropositive study participants with (*n *= 180) and without (*n *= 306) CHD (Table S4). Interestingly, percentages of T‐cell subpopulations in the CD4 and CD8 were not associated with CHD prevalence. However, inflammation markers were different: Basal TNF‐α serum levels and leukocyte interleukin‐6 responses to LPS stimulation were higher in CMV‐seropositive patients with CHD (Table S4).

**Figure 1:**
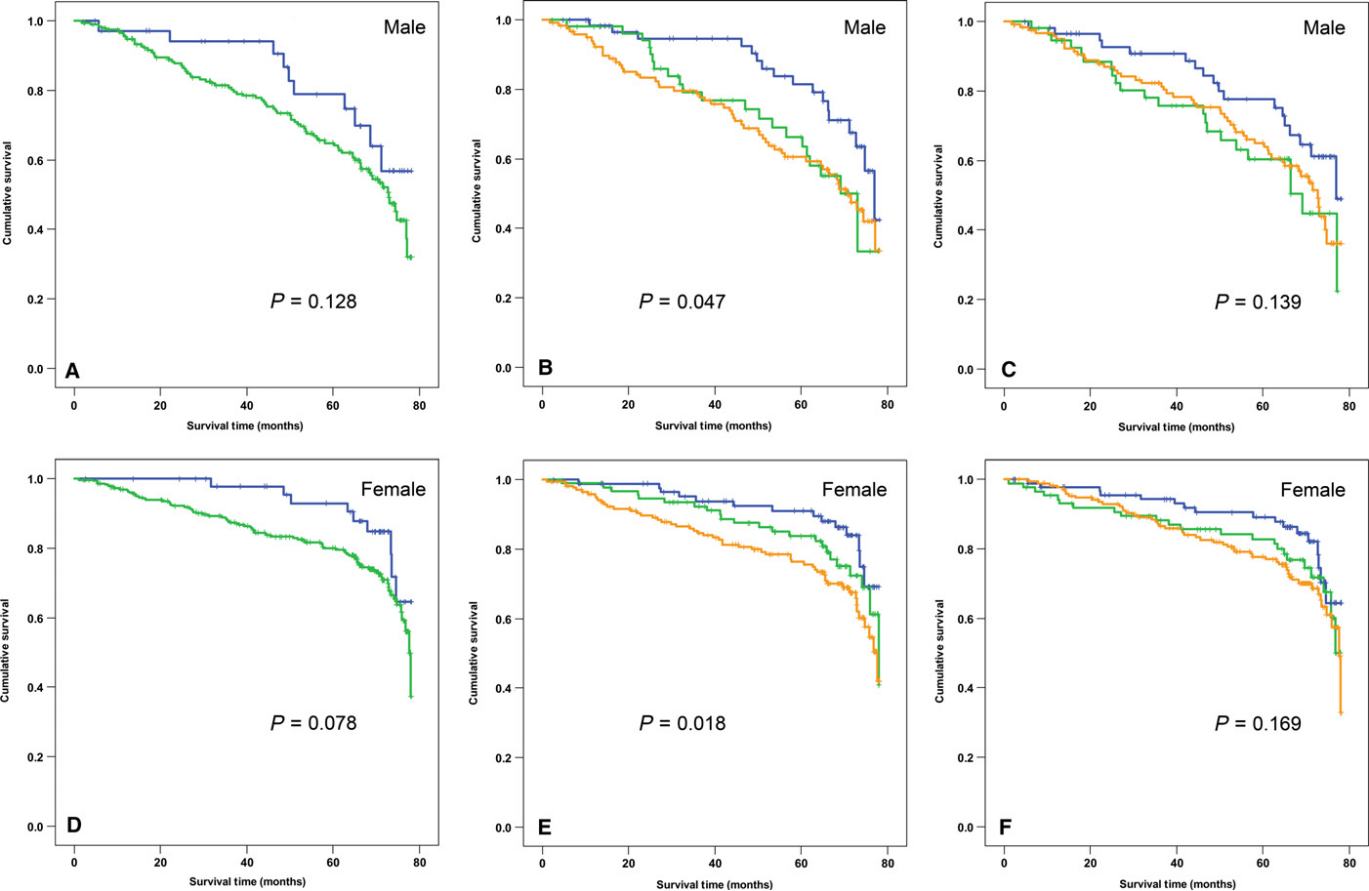
Association of cardiovascular mortality with CMV status and T‐cell senescence in men and women. Kaplan–Meier survival curves for men (A–C) and women (D–F). A, D. Survival and CMV serostatus. Blue: CMV‐seronegative participants, green: CMV‐seropositive participants. B, E Survival and senescence‐like CD4 effector memory cells (CD4^+^ CD45^RO^ ^+^ CD27^−^ CD28^−^). Blue: lowest quartile, green highest quartile, brown intermediate quartiles 2 and 3 combined as reference. C, F Survival and ratio of near‐senescent (CD3+ CD27^−^) to nonsenescent (CD3^+^ CD27^+^) CD8 T cells. Colors as for B and E. *P* values (log‐rank test) are indicated, *n *= 237 (males) and 357 (females).

Finally, survival analyses were repeated for mortality by cardiovascular causes only. There were 134 deaths from myocardial infarction (MI) or stroke (I20‐25 or I60‐69) and 184 deaths from any cardiovascular disease (I00‐69). In gender‐adjusted log‐rank analyses, positive CMV status was significantly associated with increased six‐year cardiovascular mortality (HR 1.75, 95% CI 1.09–2.82, *P* = 0.021) and increased death from MI and stroke (HR 1.89, 95% CI 1.07–3.36, *P* = 0.029). However, these associations were no longer significant in separate analyses for men and women (Fig. 1A and D). The CD4/CD8 ratio was not associated with cardiovascular mortality in our cohort (Table S5). A low percentage of senescence‐like CD4 T cells (CD4^+^CD45^RO+^CD27^−^CD28^−^) predicted decreased cardiovascular mortality (HR 0.48 [0.32–0.72], *P* < 0.001, adjusted for gender, Table S5), which was confirmed by separate analyses in men and women (Fig. 1B and E). Moreover, a low percentage of nonsenescent CD8 memory T cells (CD3^+^CD8^+^CD45^RO+^CD27^+^) predicted increased cardiovascular mortality (HR 1.78 [1.27–2.48], *P* = 0.001) in the Cox regression model after adjusting for gender (Table S5). It is known that during progression to immunosenescence CD8 cells lose the CD28 coreceptor first followed by CD27 loss (Koch *et al*., 2008), indicating that the loss of CD27 in the CD8 population delineates a more senescent phenotype. Indeed, CD8^+^CD27^−^ T cells, even if they are still capable of proliferation, show multiple characteristics of a senescent phenotype, including telomere dysfunction and activation of p38 MAPK signaling (Henson *et al*., 2009; van de Berg *et al*., 2010; Lanna *et al*., 2013). Even if they retain long telomeres, CD8+CD27‐ T cells show activated DNA damage responses, specifically persistent DNA damage response associated with dysfunctional telomeres, and mitochondrial dysfunction (Fig. S1), two major characteristics of the senescent phenotype (Passos *et al*., 2010; Jurk *et al*., 2014). Accordingly, a low ratio of near‐senescent (CD27^−^) to nonsenescent (CD27^+^) CD8T cells also predicted decreased cardiovascular mortality (HR 0.60 [0.41–0.88], *P* = 0.01, adjusted for gender, Table S5) although these associations did not remain significant in separate log‐rank analyses for men and women (Fig. 1C and F). Associations of both senescence‐like CD4 cells and nonsenescent CD8 TCM cells with cardiovascular mortality remained significant after adjustment for CMV status (Table S5); however, after further adjustment for comorbidity, a lower number of senescence‐like CD4 T cells were the only parameter to remain indicative of a favorable outcome. This remained so after further adjusting for all inflammation markers (HR 0.57 [0.34–0.94], *P* = 0.027). Likewise, a low number of senescence‐like CD4 T cells also predicted decreased risk of death from MI and stroke specifically after adjusting for gender, CMV, disease count, and cytokines (HR 0.62 [0.40–0.95], *P* = 0.027).

Earlier studies had shown the impact of an ‘immune risk phenotype’ (IRP), a simplified surrogate parameter for an aged immune system (immunosenescence) associated with CMV infection and a low CD4/CD8 ratio, on mortality in octa‐ and nonagenarians (Wikby *et al*., 2002; Nilsson *et al*., 2003). In our population, these parameters were not significant predictors of all‐cause mortality, but CMV (although not the Cd4/CD8 ratio) predicted cardiovascular mortality as well as death from MI or stroke in agreement with recent results (Savva *et al*., 2013). Our data specify the alterations caused by CMV infection in the lymphocyte compartment that are driving adverse events as those related to cell senescence in the CD4 and CD8 T‐cell compartments. Cell senescence, including immune cell senescence, is associated with telomere dysfunction, MAP kinase activation and mitochondrial dysfunction (Henson *et al*., 2009; van de Berg *et al*., 2010; Passos *et al*., 2010; Lanna *et al*., 2013; Jurk *et al*., 2014), which in turn cause hyperactivation of NF‐κB‐ and C/EBPβ‐driven secretion of pro‐inflammatory peptides (the so‐called senescence‐associated secretory phenotype or SASP (Coppe *et al*., 2008)). Conversely, chronic activation of inflammatory signaling can aggravate the senescent phenotype (Jurk *et al*., 2014) and thus contribute to vascular dysfunction and CVD (Wang & Bennett, 2012). Thus, our data support a possible causal role for immunosenescence in CHD and are well in accord with the idea of a generalized chronic pro‐inflammatory state as a risk factor for the disease. This possible cell‐based mechanistic link between CMV and CHD via ongoing generation of highly pro‐inflammatory T cells of senescent phenotype could also explain differences in the progression of age‐related atherosclerosis between individuals.

Our study was limited as we could not exclude subclinical CHD in the remaining CMV+ patients, nor was the diagnosis of CHD angiographically confirmed. It should also be noted that some studies have doubted a role for CMV in immunosenescence and aging‐associated inflammation (Cicin‐Sain *et al*., 2011; Bartlett *et al*., 2012). However, one of them used a rhesus macaque model of aging (Cicin‐Sain *et al*., 2011), where old was defined as up to 26 years, and probably not comparable with the age of our octogenarians. The study by Bartlett took CMV seropositivity into consideration, but not T‐cell immunity specifically (Bartlett *et al*., 2012). Derhovassian and coworkers have shown that the response to CMV can be very heterogeneous and that immunological control of the virus can contribute to decreased mortality rate (Derhovanessian *et al*., 2010). Our results suggest CMV seropositivity as a driver of T‐cell senescence that at least in the CD4 compartment independently predicts cardiovascular mortality.

## Funding

The study was supported by the Medical Research Council (G0500997 to TK, CJ and TvZ, G0601333 to TvZ) and the NIHR Biomedical Research Centre in Ageing and Chronic Disease.

## Conflict of interest

None declared.

## Supporting information


**Data S1.** Experimental Procedures.
**Table S1.** CMV‐dependent differences in laboratory results (*n *= 749).
**Table S2.** Gender‐related differences in laboratory results (CMV‐positive participants only).
**Table S3.** All‐cause mortality (all participants with known CMV status).
**Table S4.** CHD‐related differences in laboratory results (CMV‐positive participants only).
**Table S5.** Cardiovascular mortality (all participants with known CMV status).
**Fig. S1.** A senescence‐like phenotype in CD8^+^CD56^−^CD27^−^ T cells.
**Fig. S2.** Examples for the gating strategies for CD4 (A) and CD8 (B) T cells.
