# Supplementary material for: CMV seropositivity and T‐cell senescence predict increased cardiovascular mortality in octogenarians: results from the Newcastle 85+ study
Source: Aging Cell. 2015 Dec 22;15(2):389–92. doi: 10.1111/acel.12430 (PMC4783336; doi:10.1111/acel.12430)
Supplement: Supplementary file 1 — Data S1. Experimental Procedures. Table S1. CMV‐dependent differences in laboratory results (n = 749). Table S2. Gender‐related differences in laboratory results (CMV‐positive participants only). Table S3. All‐cause mortality (all participants with known CMV status). Table S4. CHD‐related differences in laboratory results (CMV‐positive participants only). Table S5. Cardiovascular mortality (all participants with known CMV status). Fig. S1. A senescence‐like phenotype in CD8+CD56−CD27− T cells. Fig. S2. Examples for the gating strategies for CD4 (A) and CD8 (B) T cells. [file ACEL-15-389-s001.docx]

**Experimental Procedures**

**Study population**

The methodology for the Newcastle 85+ Study has been reported (Collerton *et al.*, 2007; Collerton *et al.*, 2009). In brief, members of the 1921 birth cohort living in Newcastle or North Tyneside (North-East England) were recruited at around age 85 through general practice patient lists. People living in institutions and those with cognitive impairment were included. At baseline the Newcastle 85+ cohort was socio-demographically representative of the local population and of England and Wales (Collerton *et al.*, 2009). The research complied with the requirements of the Declaration of Helsinki. Ethical approval was obtained from the Newcastle and North Tyneside 1 Research Ethics Committee (reference number 06/Q0905/2); written informed consent was obtained from participants and where people lacked capacity to consent, for example because of dementia, a formal written opinion was sought from a relative or carer.

**Blood-based biomarkers**

Biomarkers were measured from a blood sample drawn between 7 am and 10.30 am following an 8h overnight fast and delivered to the laboratory for initial processing within one hour of draw. Blood samples were collected within 6 months of participant assessment with the exception of cytomegalovirus (CMV) status where the timeframe was within 18 months (Martin-Ruiz *et al.*, 2011).

***Lymphocyte immunophenotyping:*** Blood samples were analysed by 4-colour flow-cytometry (Becton Dickinson FACScan Flow Cytometer) using fluorescence-labelled antibodies from BD Biosciences (Oxford, UK). Lymphocyte compartments were defined by the following marker combinations: **B** cells CD19^+^, **T** cells CD3^+^, **CD4** T cells CD3^+^CD4^+^, **CD8** T cells CD3^+^CD8^+^, **CD4** naïve CD4^+^CD45^RO-^CD27^+^, **CD4** central memory (T**_CM_**) CD4^+^CD45^RO+^CD27^+^, **CD4** effector memory (T**_EM_**) CD4^+^CD45^RO+^CD27^-^, **CD4** senescence-like T**_EM_** CD4^+^CD45^RO+^CD27^-^CD28^-^, **CD8** naïve CD3^+^CD8^+^CD45^RO-^CD27^+^, **CD8** T**_CM_** CD3^+^CD8^+^CD45^RO+^CD27^+^, **CD8** T**_EM_** CD3^+^CD8^+^CD45^RO+^CD27^-^, **CD8** senescence-like effector memory (T**_EMRA_**) CD3^+^CD8^+^CD45^RO-^CD27^-^. The gating strategy is exemplified in suppl. Fig. S2. Our definition of senescence in T cells was based on multiple studies that used the lack of CD27 expression as an indicator for senescent CD8 T**_EM_** and the lack of CD28 for senescent CD4 T**_EM_** cells, owing to the fact that CD4 and CD8 T cells differ in the sequence in that they lose their co-receptors. Immunophenotyping CVs were between 2.4% (CD4+ T cells) and 9.8% (CD4^+^CD45^RO+^CD27^-^CD28^-^ cells).

***CMV serostatus:*** CMV IgG concentration was measured using a commercial enzyme linked fluorescent assay (bioMerieux VIDAS, bioMerieux SA, France); assay sensitivity 99.2% and specificity 100%. A value of less than 4 arbitrary units was interpreted as negative and of 6 or more as positive. A value less than 6 but greater than or equal to 4 arbitrary units was regarded as equivocal; no study samples fell in the equivocal range.

***High-sensitivity CRP*:** assessed by Dade Behring CardioPhase hsCRP immunoassay.

***IL6 and TNF-alpha:*** Basal and lipopolysaccharide (Invivogen Ultrapure LPS) stimulated production of IL-6 and TNF-α by peripheral blood mononuclear cells (PBMC) was measured. Lithium heparin blood samples were stimulated for cytokine production with Invivogen Ultrapure LPS (Autogenbioclear, Nottingham, UK) and after 24h incubation at 37˚C serum supernatants were collected. Basal and post-stimulation levels of IL-6 and TNF-α were assessed by electrochemiluminescence on a 96-well Multi-SPOT Meso Scale Discovery assay with a SECTOR Imager 6000 (Meso Scale Diagnostics, LLC. Gaithersburg, MD, USA).

**Measurement of senescence markers in CD8 T cells:** CD8+CD56+, CD8+CD56-CD27+ and CD8+CD56-CD27- T cells were sorted from 40 ml of blood (EDTA S-Monovette 9 ml, Cat. No. 02-1066-001, Sarstedt, Nümbrecht, Germany) from 4 healthy volunteers. Peripheral blood mononuclear cells (PBMCs) were obtained after density gradient centrifugation using Ficoll-Hypaque (Cat. No. L6115, Biochrom, Germany). After two washes with PBS, the PBMCs were resuspended in the ice chilled MACS buffer (Miltenyi) containing PBS with 0.5 % FCS and 2 mM EDTA (Tritriplex III 1.1 %). The cell fractionation strategy was based on immunomagnetic sorting (MACS, Miltenyi Biotec, Bergisch Gladbach, Germany). All separation steps were performed by using LS columns (Cat. No. 130-042-401) placed in the QuadroMACS Separator (MACS MultiStand Cat. No. 130-042-303). Briefly, CD8+ cells were first isolated from PBMCs with the CD8 Multisort Kit (Cat. No. 552-01). After removal of CD8-Beads from the cell surface, the CD8+ cells were depleted from NK cells by using CD56 MicroBeads (Cat. No. 130-050-401). The negatively selected CD8+CD56- cells were highly purified cytotoxic T cells as confirmed by flow cytometry (percentage of CD3+CD8+ over 98%). For further isolation of near-senescent CD8 T cell subpopulations we subsequently sorted them into CD27+ and CD27- cells using Anti-Biotin Microbeads (Cat. No. 130-051-601). Purity of CD8+CD27+CD56- and CD8+CD27-CD56- cells was over 95%. Sorted cells were divided into two aliquots, one was used for measurement of telomere length by qPCR as described (Martin-Ruiz et al. 2011). Cells in the other aliquot were used for measurement of telomere-associated DNA damage foci by immunoFISH (Hewitt et al. 2012) and of cellular superoxide levels by dihydroethidine staining and mitochondrial mass by Mitotracker Green staining followed by flow cytometric measurement of fluorescence intensity as described (Passos et al. 2010).

**Morbidity, mortality and causes of death**

A multidimensional health assessment was carried out in the participant's usual residence by a research nurse. Data on pre-existing diseases and prescribed medication were obtained from general practice medical records. Date and cause of death were obtained through the UK Health and Social Care Information Service. Survival time (in years) was calculated from date of blood draw to date of death or censored at 31 December 2013 (mean follow-up 65 months, overall survival rate 47.3%). We defined cardiovascular death as ICD codes I00-I69 and death due to myocardial infarction or stroke to be I20-I25 and I60-I69.

**Statistics**

We compared the baseline socio-demographic, health, lifestyle factors and T-cell distribution of participants who were CMV positive and negative by Chi-square tests (for binary and nominal variables) and Mann-Whitney U tests (for ordinal variables), presenting percentages in each category in the former and median and interquartile range (IQR) in the latter. Additionally we compared the T-cell distribution of CMV seropositive participants firstly with and without CHD and then between men and women, by Mann-Whitney U tests. We assessed the association of CMV status and leucocyte subsets on survival initially by log-rank tests and then by fitting Cox proportional hazards regression models adjusted as follows for: gender; gender, CMV status; gender, CMV status, disease count; gender, CMV status, disease count, TNFα, IL6, CD8 T**_EM_**. Only those participants (n=594) with complete data for all leukocyte parameters, CMV status and all confounding factors (comorbidity, basal interleukin-6 and TNF-α) were analysed. The overall survival rate for this analysed sample was 49.3%.

References

Collerton, J, Barrass, K, Bond, J, Eccles, M, Jagger, C, James, O, Martin-Ruiz, C, Robinson, L, von Zglinicki, T, Kirkwood, T (2007) The Newcastle 85+ study: biological, clinical and psychosocial factors associated with healthy ageing: study protocol. *BMC Geriatr* **7**, 14.

Collerton, J, Davies, K, Jagger, C, Kingston, A, Bond, J, Eccles, MP, Robinson, LA, Martin-Ruiz, C, von Zglinicki, T, James, OF, Kirkwood, TB (2009) Health and disease in 85 year olds: baseline findings from the Newcastle 85+ cohort study. *BMJ* **339**, b4904.

Hewitt G, Jurk D, Marques FD, Correia-Melo C, Hardy T, Gackowska A, Anderson R, Taschuk M, Mann J, Passos JF (2012) Telomeres are favoured targets of a persistent DNA damage response in ageing and stress-induced senescence. *Nat Commun.* 3:708.

Martin-Ruiz, C, Jagger, C, Kingston, A, Collerton, J, Catt, M, Davies, K, Dunn, M, Hilkens, C, Keavney, B, Pearce, SH, den Elzen, WP, Talbot, D, Wiley, L, Bond, J, Mathers, JC, Eccles, MP, Robinson, L, James, O, Kirkwood, TB, von Zglinicki, T (2011) Assessment of a large panel of candidate biomarkers of ageing in the Newcastle 85+ study. *Mech Ageing Dev* **132**, 496-502.

Passos, JF, Nelson, G, Wang, C, Richter, T, Simillion, C, Proctor, C, Miwa, S, Olijslagers, S, Hallinan, J, Wipat, A, Saretzki, G, Kirkwood, T, Rudolph, KL, von Zglinicki, T (2010) Feedback between p21 and reactive oxygen production is necessary for cell senescence. *Molecular Systems Biol,* 6: 347.

**Supplementary tables**

**Table S1.** CMV-dependent differences in laboratory results (n=749)

| **Parameter** | **CMV negative (n=108)** | | **CMV positive (n=641)** | | **p-Values** |
| --- | --- | --- | --- | --- | --- |
|  | Median | IQR | Median | IQR |  |
| White blood cells (x10^9^/l) | 6.30 | 5.05 - 7.65 | 6.40 | 5.4 - 7.6 | 0.421 |
| Neutrophils (x10^9^/l) | 3.82 | 2.78 - 4.82 | 3.71 | 2.86 - 4.48 | 0.471 |
| Monocytes (x10^9^/l) | 0.53 | 0.46 - 0.65 | 0.53 | 0.44 - 0.66 | 0.604 |
| Lymphocytes (x10^9^/l) | 1.57 | 1.16 - 1.96 | 1.86 | 1.48 - 2.29 | <0.001 |
| B-cells (%) | 8.40 | 6.02 - 13.18 | 7.38 | 4.93 - 10.7 | 0.009 |
| T-cells (%) | 66.55 | 59.15 - 74.2 | 64.90 | 58.05 - 71.4 | 0.162 |
| % CD4 | 51.30 | 42.5 - 59 | 42.00 | 34.5 - 51 | <0.001 |
| % CD8 | 12.13 | 7.9 - 17.25 | 23.90 | 16.03 - 33.56 | <0.001 |
| CD4/CD8 ratio | 4.14 | 2.65 - 7.19 | 1.74 | 1.08 - 2.94 | <0.001 |
| CD4 Naïve (% CD4) | 48.10 | 34.1 - 60.1 | 38.50 | 25.3 - 51.5 | <0.001 |
| CD4 T_CM_ (% CD4) | 45.20 | 33.3 - 56.5 | 41.40 | 33.1 - 51.2 | 0.041 |
| CD4 T_EM_ (% CD4) | 4.41 | 2.74 - 7.06 | 12.50 | 7.1 - 19.6 | <0.001 |
| Senescence-like CD4 T_EM_ (% CD4 T_EM_) | 4.46 | 2.17 - 8.44 | 41.10 | 21.8 - 62.4 | <0.001 |
| CD4 T_EM_ / Naïve ratio | 0.10 | 0.05 - 0.2 | 0.33 | 0.15 - 0.7 | <0.001 |
| CD8 Naïve (% CD8) | 26.96 | 19.1 - 36.33 | 14.10 | 8.05 - 21.95 | <0.001 |
| CD8 T_CM_ (% CD8) | 50.50 | 41.48 - 60.15 | 29.06 | 20.01 - 40.91 | <0.001 |
| CD8 T_EM_ (% CD8) | 7.04 | 3.84 - 11.26 | 18.37 | 10.06 - 29.64 | <0.001 |
| CD8 T_EMRA_ (% CD8) | 6.73 | 2.58 - 13.75 | 28.09 | 15.83 - 40.15 | <0.001 |
| IL-6, basal (ng/ml) stimulated | 19736 | 9847 - 30400 | 22402 | 11775 - 32141 | 0.241 |
| IL-6, basal (ng/ml) unstimulated | 11.80 | 2.53 - 93.93 | 15.12 | 4.16 - 159.17 | 0.159 |
| TNF-alpha, basal (pg/ml) | 437 | 225 - 832 | 509 | 233 - 993 | 0.210 |
| CRP (mg/l) | 2.20 | 0.9 - 5 | 2.70 | 1.3 - 6.2 | 0.074 |

| **Table S2.** Gender-related differences in laboratory results (CMV-positive participants only) | | | | | |  |
| --- | --- | --- | --- | --- | --- | --- |
| **Parameter** | **men (N=232)** | | **women (N=373)** | | **p-Values** | |
|  | Median | IQR | Median | IQR |  |  |
| White blood cells (x10^9^/l) | 6.60 | 5.72 - 7.78 | 6.30 | 5.35 - 7.5 | 0.036 | |
| Neutrophils (x10^9^/l) | 3.80 | 2.98 - 4.54 | 3.64 | 2.82 - 4.46 | 0.174 | |
| Monocytes (x10^9^/l) | 0.58 | 0.45 - 0.72 | 0.51 | 0.42 - 0.62 | <0.001 | |
| Lymphocytes (x10^9^/l) | 1.84 | 1.45 - 2.23 | 1.89 | 1.49 - 2.31 | 0.346 | |
| B-cells (%) | 6.00 | 3.58 - 9.3 | 8.26 | 5.8 - 11.4 | <0.001 | |
| T-cells (%) | 63.50 | 54.9 - 69.75 | 65.65 | 59.7 - 71.8 | 0.001 | |
| % CD4 | 37.80 | 30.65 - 46.03 | 44.50 | 37.65 - 52.85 | <0.001 | |
| % CD8 | 26.18 | 17.3 - 36.6 | 23.06 | 15.46 - 32.19 | 0.002 | |
| CD4/CD8 ratio | 1.41 | 0.91 - 2.53 | 1.97 | 1.23 - 3.47 | <0.001 | |
| CD4 Naïve (% CD4) | 32.80 | 21.68 - 46.9 | 41.50 | 29.25 - 53.25 | <0.001 | |
| CD4 T_CM_ (% CD4) | 43.50 | 32.97 - 52.93 | 40.40 | 33.05 - 49.65 | 0.051 | |
| CD4 T_EM_ (% CD4) | 13.65 | 7.81 - 23.78 | 12.00 | 6.77 - 18.5 | 0.018 | |
| Senescence-like CD4 T_EM_ | 37.80 | 21.3 - 61.1 | 43.75 | 22.35 - 63.85 | 0.143 | |
| (% CD4 T_EM_) |  |  |  |  |  |  |
| CD4 T_EM_ / Naïve ratio | 0.43 | 0.18 - 0.97 | 0.29 | 0.15 - 0.58 | 0.001 | |
| CD8 Naïve (% CD8) | 11.55 | 6.7 - 19.99 | 15.17 | 9.41 - 23.15 | 0.001 | |
| CD8 T_CM_ (% CD8) | 28.75 | 19.6 - 39.61 | 29.83 | 20.33 - 42.1 | 0.270 | |
| CD8 T_EM_ (% CD8) | 21.05 | 10.77 - 31.25 | 17.19 | 9.55 - 27.7 | 0.046 | |
| CD8 T_EMRA_ (% CD8) | 27.59 | 16.06 - 41.91 | 28.13 | 15.69 - 39.75 | 0.785 | |
| IL-6, basal (ng/ml) stimulated | 23939.80 | 13733.47 - 33748.66 | 21602.30 | 11040.7 - 31396.91 | 0.060 | |
| IL-6, basal (ng/ml) unstimulated | 17.46 | 4.55 - 208.59 | 14.31 | 3.87 - 127.84 | 0.209 | |
| TNF-alpha, basal (pg/ml) | 552.73 | 288.76 - 1104.69 | 484.27 | 225.12 - 930.22 | 0.137 | |
| CRP (mg/l) | 2.80 | 1.2 - 6.4 | 2.60 | 1.3 - 5.8 | 0.335 | |

**Table S3.** All-cause mortality (all participants with known CMV status)

|  | ***Model 1: Adjusted for Sex*** | | | |  | ***Model 2: Adjusted for Sex, CMV*** | | | |
| --- | --- | --- | --- | --- | --- | --- | --- | --- | --- |
| Variable | **HR** | **[95% CI]** | | **P-Value** |  | **HR** | **[95% CI]** | | **P-Value** |
| **CD4 T cells** |  |  |  | 0.217 |  |  |  |  | 0.228 |
| Q2 & Q3 Combined (Reference) | 1.00 |  |  |  |  | 1.00 |  |  |  |
| Lower Quartile | 1.07 | 0.82 | 1.39 | 0.639 |  | 1.05 | 0.80 | 1.37 | 0.716 |
| Upper Quartile | 0.81 | 0.60 | 1.08 | 0.152 |  | 0.80 | 0.60 | 1.08 | 0.144 |
| **Total CD8 (CD3^+^CD8^+^)** |  |  |  | 0.821 |  |  |  |  | 0.727 |
| Q2 & Q3 Combined (Reference) | 1.00 |  |  |  |  | 1.00 |  |  |  |
| Lower Quartile | 0.98 | 0.74 | 1.28 | 0.861 |  | 1.04 | 0.78 | 1.39 | 0.781 |
| Upper Quartile | 0.91 | 0.69 | 1.21 | 0.530 |  | 0.92 | 0.69 | 1.21 | 0.535 |
| **CD4/CD8** |  |  |  | 0.186 |  |  |  |  | 0.267 |
| Upper | 0.82 | 0.62 | 1.10 | 0.186 |  | 0.85 | 0.63 | 1.14 | 0.267 |
| **CD4 Naïve (CD4^+^CD45^RO-^CD27^+^)** |  |  |  | 0.368 |  |  |  |  | 0.444 |
| Q2 & Q3 Combined (Reference) | 1.00 |  |  |  |  | 1.00 |  |  |  |
| Lower Quartile | 1.14 | 0.87 | 1.51 | 0.336 |  | 1.12 | 0.85 | 1.48 | 0.420 |
| Upper Quartile | 0.91 | 0.69 | 1.21 | 0.515 |  | 0.91 | 0.69 | 1.21 | 0.510 |
| **CD4 T_CM_ ( CD4^+^CD45^RO+^CD27^+^)** |  |  |  | 0.067 |  |  |  |  | 0.071 |
| Q2 & Q3 Combined (Reference) | 1.00 |  |  |  |  | 1.00 |  |  |  |
| Lower Quartile | 1.18 | 0.91 | 1.54 | 0.212 |  | 1.18 | 0.91 | 1.54 | 0.218 |
| Upper Quartile | 0.81 | 0.61 | 1.08 | 0.159 |  | 0.81 | 0.61 | 1.09 | 0.161 |
| **CD4 T_EM_ (CD4^+^CD45^RO+^CD27^-^)** |  |  |  | 0.354 |  |  |  |  | 0.557 |
| Q2 & Q3 Combined (Reference) | 1.00 |  |  |  |  | 1.00 |  |  |  |
| Lower Quartile | 0.87 | 0.66 | 1.16 | 0.346 |  | 0.91 | 0.67 | 1.23 | 0.531 |
| Upper Quartile | 1.11 | 0.84 | 1.46 | 0.470 |  | 1.10 | 0.83 | 1.45 | 0.505 |
| **CD4 Senescence-like T_EM_ (CD4^+^CD45^RO+^CD27^-^CD28^-^)** | |  |  | 0.042 |  |  |  |  | 0.084 |
| Q2 & Q3 Combined (Reference) | 1.00 |  |  |  |  | 1.00 |  |  |  |
| Lower Quartile | 0.69 | 0.52 | 0.92 | 0.012 |  | 0.68 | 0.48 | 0.96 | 0.028 |
| Upper Quartile | 0.89 | 0.67 | 1.17 | 0.399 |  | 0.89 | 0.67 | 1.17 | 0.401 |
| **Memory/Naïve CD4** |  |  |  | 0.219 |  |  |  |  | 0.348 |
| Q2 & Q3 Combined (Reference) | 1.00 |  |  |  |  | 1.00 |  |  |  |
| Lower Quartile | 0.80 | 0.60 | 1.06 | 0.113 |  | 0.82 | 0.61 | 1.10 | 0.182 |
| Upper Quartile | 1.03 | 0.78 | 1.35 | 0.860 |  | 1.02 | 0.78 | 1.34 | 0.884 |
| **CD8 Naïve (CD3^+^CD8^+^CD45^RO-^CD27^+^)** | |  |  | 0.794 |  |  |  |  | 0.708 |
| Q2 & Q3 Combined (Reference) | 1.00 |  |  |  |  | 1.00 |  |  |  |
| Lower Quartile | 0.93 | 0.70 | 1.23 | 0.599 |  | 0.90 | 0.68 | 1.19 | 0.449 |
| Upper Quartile | 0.92 | 0.70 | 1.22 | 0.558 |  | 0.92 | 0.70 | 1.22 | 0.560 |
| **CD8 T_CM_ (CD3^+^CD8^+^CD45^RO+^CD27^+^)** |  |  |  | 0.088 |  |  |  |  | 0.139 |
| Q2 & Q3 Combined (Reference) | 1.00 |  |  |  |  | 1.00 |  |  |  |
| Lower Quartile | 1.34 | 1.02 | 1.74 | 0.033 |  | 1.30 | 0.99 | 1.71 | 0.059 |
| Upper Quartile | 1.03 | 0.78 | 1.37 | 0.816 |  | 1.02 | 0.77 | 1.36 | 0.891 |
| **CD8 T_EM_ (CD3^+^CD8^+^CD45^RO+^CD27^-^)** |  |  |  | 0.181 |  |  |  |  | 0.243 |
| Q2 & Q3 Combined (Reference) | 1.00 |  |  |  |  | 1.00 |  |  |  |
| Lower Quartile | 1.00 | 0.76 | 1.33 | 0.964 |  | 1.06 | 0.79 | 1.42 | 0.709 |
| Upper Quartile | 1.27 | 0.97 | 1.67 | 0.081 |  | 1.26 | 0.96 | 1.66 | 0.094 |
| **CD8 T_EMRA_ (CD3^+^CD8^+^CD45^RO-^CD27^-^)** |  |  |  | 0.218 |  |  |  |  | 0.400 |
| Q2 & Q3 Combined (Reference) | 1.00 |  |  |  |  | 1.00 |  |  |  |
| Lower Quartile | 0.79 | 0.60 | 1.05 | 0.099 |  | 0.81 | 0.60 | 1.11 | 0.190 |
| Upper Quartile | 1.00 | 0.76 | 1.32 | 0.982 |  | 1.00 | 0.76 | 1.32 | 0.993 |

Highlighted fields remain significant after correction for multiple testing

| **Table S4.** CHD-related differences in laboratory results (CMV-positive participants only)   \| **Parameter** \| **No CHD (n=306)** \| \| **CHD (n=180)** \| \| **p-Values** \| \| --- \| --- \| --- \| --- \| --- \| --- \| \| Median \| IQR \| Median \| IQR \| \| White blood cells (x10^9^/l) \| 6.30 \| 5.38 - 7.5 \| 6.60 \| 5.6 - 7.9 \| 0.029 \| \| Neutrophils (x10^9^/l) \| 3.60 \| 2.81 - 4.41 \| 3.92 \| 2.99 - 4.6 \| 0.029 \| \| Monocytes (x10^9^/l) \| 0.53 \| 0.43 - 0.64 \| 0.55 \| 0.45 - 0.68 \| 0.050 \| \| Lymphocytes (x10^9^/l) \| 1.86 \| 1.5 - 2.3 \| 1.84 \| 1.48 - 2.29 \| 0.935 \| \| B-cells (%) \| 7.04 \| 4.82 - 10.4 \| 7.68 \| 5.1 - 11.6 \| 0.037 \| \| T-cells (%) \| 65.20 \| 58.4 - 71.4 \| 64.40 \| 57.5 - 71.9 \| 0.728 \| \| % CD4 \| 41.90 \| 35.5 - 50.8 \| 42.80 \| 33.65 - 51.18 \| 0.754 \| \| % CD8 \| 23.68 \| 15.98 - 33.47 \| 24.02 \| 15.51 - 33.13 \| 0.994 \| \| CD4/CD8 ratio \| 1.78 \| 1.09 - 2.88 \| 1.73 \| 1.08 - 3.25 \| 0.852 \| \| CD4 Naïve (% CD4) \| 38.10 \| 26 - 51.5 \| 39.00 \| 23.82 - 51.18 \| 0.959 \| \| CD4 T_CM_ (% CD4) \| 41.90 \| 33 - 52 \| 40.90 \| 32.9 - 50.5 \| 0.352 \| \| CD4 T_EM_ (% CD4) \| 12.80 \| 7.34 - 18.6 \| 12.05 \| 6.91 - 21.75 \| 0.819 \| \| Senescence-like CD4 T_EM_ \| 40.05 \| 21.8 - 61.03 \| 45.50 \| 22.2 - 65.3 \| 0.093 \| \| (% CD4 T_EM_) \| \| CD4 T_EM_ / Naïve ratio \| 0.34 \| 0.16 - 0.65 \| 0.31 \| 0.15 - 0.83 \| 0.997 \| \| CD8 Naïve (% CD8) \| 14.87 \| 7.8 - 22.22 \| 13.58 \| 8.41 - 21.09 \| 0.283 \| \| CD8 T_CM_ (% CD8) \| 30.08 \| 20.91 - 42.28 \| 27.84 \| 19.31 - 38.96 \| 0.143 \| \| CD8 T_EM_ (% CD8) \| 17.78 \| 9.76 - 28.69 \| 18.94 \| 10.44 - 30.94 \| 0.245 \| \| CD8 T_EMRA_ (% CD8) \| 27.12 \| 15.36 - 39.91 \| 29.02 \| 16.58 - 40.56 \| 0.362 \| \| IL-6, basal (ng/ml) stimulated \| 20198 \| 11113 - 30868 \| 25857 \| 12478 - 33478 \| 0.005 \| \| IL-6, basal (ng/ml) unstimulated \| 14.9 \| 4.2 - 143.3 \| 16.0 \| 3.9 - 176.9 \| 0.644 \| \| TNF-alpha, basal (pg/ml) \| 477 \| 226 - 834 \| 629 \| 255 - 1152 \| 0.010 \| \| CRP (mg/l) \| 2.60 \| 1.2 - 5.85 \| 2.70 \| 1.4 - 6.7 \| 0.296 \| |
| --- | --- | --- | --- | --- | --- | --- | --- | --- | --- | --- | --- | --- | --- | --- | --- | --- | --- | --- | --- | --- | --- | --- | --- | --- | --- | --- | --- | --- | --- | --- | --- | --- | --- | --- | --- | --- | --- | --- | --- | --- | --- | --- | --- | --- | --- | --- | --- | --- | --- | --- | --- | --- | --- | --- | --- | --- | --- | --- | --- | --- | --- | --- | --- | --- | --- | --- | --- | --- | --- | --- | --- | --- | --- | --- | --- | --- | --- | --- | --- | --- | --- | --- | --- | --- | --- | --- | --- | --- | --- | --- | --- | --- | --- | --- | --- | --- | --- | --- | --- | --- | --- | --- | --- | --- | --- | --- | --- | --- | --- | --- | --- | --- | --- | --- | --- | --- | --- | --- | --- | --- | --- | --- | --- | --- | --- | --- | --- | --- | --- | --- | --- | --- | --- | --- | --- | --- | --- | --- | --- | --- | --- | --- | --- |

**Table S5.** Cardiovascular mortality (all participants with known CMV status)

|  | ***Model 1: Adjusted for Sex*** | | | |  | ***Model 2: Adjusted for Sex, CMV*** | | | |
| --- | --- | --- | --- | --- | --- | --- | --- | --- | --- |
| Variable | **HR** | **[95% CI]** | | **P-Value** |  | **HR** | **[95% CI]** | | **P-Value** |
| **CD4 T cells** |  |  |  | 0.751 |  |  |  |  | 0.757 |
| Q2 & Q3 Combined (Reference) | 1.00 |  |  |  |  | 1.00 |  |  |  |
| Lower Quartile | 1.00 | 0.71 | 1.42 | 0.993 |  | 0.97 | 0.69 | 1.37 | 0.868 |
| Upper Quartile | 0.88 | 0.61 | 1.26 | 0.479 |  | 0.87 | 0.60 | 1.26 | 0.459 |
| **Total CD8 (CD3^+^CD8^+^)** |  |  |  | 0.545 |  |  |  |  | 0.952 |
| Q2 & Q3 Combined (Reference) | 1.00 |  |  |  |  | 1.00 |  |  |  |
| Lower Quartile | 0.82 | 0.57 | 1.17 | 0.276 |  | 0.94 | 0.65 | 1.38 | 0.768 |
| Upper Quartile | 0.96 | 0.68 | 1.37 | 0.839 |  | 0.97 | 0.68 | 1.37 | 0.849 |
| **CD4/CD8** |  |  |  | 0.840 |  |  |  |  | 0.818 |
| Upper | 0.96 | 0.66 | 1.40 | 0.840 |  | 1.05 | 0.71 | 1.54 | 0.818 |
| **CD4 Naïve (CD4^+^CD45^RO-^CD27^+^)** |  |  |  | 0.524 |  |  |  |  | 0.728 |
| Q2 & Q3 Combined (Reference) | 1.00 |  |  |  |  | 1.00 |  |  |  |
| Lower Quartile | 1.21 | 0.85 | 1.71 | 0.293 |  | 1.14 | 0.80 | 1.63 | 0.461 |
| Upper Quartile | 0.99 | 0.69 | 1.42 | 0.969 |  | 1.00 | 0.69 | 1.43 | 0.978 |
| **CD4 T_CM_ ( CD4^+^CD45^RO+^CD27^+^)** |  |  |  | 0.341 |  |  |  |  | 0.380 |
| Q2 & Q3 Combined (Reference) | 1.00 |  |  |  |  | 1.00 |  |  |  |
| Lower Quartile | 1.22 | 0.87 | 1.73 | 0.248 |  | 1.21 | 0.86 | 1.70 | 0.281 |
| Upper Quartile | 0.92 | 0.64 | 1.33 | 0.668 |  | 0.92 | 0.64 | 1.33 | 0.666 |
| **CD4 T_EM_ (CD4^+^CD45^RO+^CD27^-^)** |  |  |  | 0.606 |  |  |  |  | 0.980 |
| Q2 & Q3 Combined (Reference) | 1.00 |  |  |  |  | 1.00 |  |  |  |
| Lower Quartile | 0.87 | 0.61 | 1.24 | 0.442 |  | 1.00 | 0.69 | 1.45 | 0.993 |
| Upper Quartile | 1.07 | 0.75 | 1.53 | 0.716 |  | 1.04 | 0.72 | 1.48 | 0.851 |
| **CD4 Senescence-like T_EM_ (CD4^+^CD45^RO+^CD27^-^CD28^-^)** |  |  |  | 0.002 |  |  |  |  | 0.018 |
| Q2 & Q3 Combined (Reference) | 1.00 |  |  |  |  | 1.00 |  |  |  |
| Lower Quartile | 0.48 | 0.32 | 0.72 | <0.001 |  | 0.52 | 0.33 | 0.83 | 0.006 |
| Upper Quartile | 0.82 | 0.57 | 1.16 | 0.256 |  | 0.81 | 0.57 | 1.16 | 0.250 |
| **Memory/Naïve CD4** |  |  |  | 0.208 |  |  |  |  | 0.452 |
| Q2 & Q3 Combined (Reference) | 1.00 |  |  |  |  | 1.00 |  |  |  |
| Lower Quartile | 0.85 | 0.59 | 1.21 | 0.362 |  | 0.93 | 0.65 | 1.35 | 0.717 |
| Upper Quartile | 1.22 | 0.86 | 1.72 | 0.269 |  | 1.20 | 0.85 | 1.69 | 0.309 |
| **CD8 Naïve (CD3^+^CD8^+^CD45^RO-^CD27^+^)** |  |  |  | 0.424 |  |  |  |  | 0.432 |
| Q2 & Q3 Combined (Reference) | 1.00 |  |  |  |  | 1.00 |  |  |  |
| Lower Quartile | 0.96 | 0.67 | 1.36 | 0.803 |  | 0.88 | 0.62 | 1.26 | 0.487 |
| Upper Quartile | 0.78 | 0.54 | 1.13 | 0.195 |  | 0.79 | 0.54 | 1.14 | 0.208 |
| **CD8 T_CM_ (CD3^+^CD8^+^CD45^RO+^CD27^+^)** |  |  |  | 0.003 |  |  |  |  | 0.015 |
| Q2 & Q3 Combined (Reference) | 1.00 |  |  |  |  | 1.00 |  |  |  |
| Lower Quartile | 1.78 | 1.27 | 2.48 | 0.001 |  | 1.64 | 1.17 | 2.32 | 0.004 |
| Upper Quartile | 1.19 | 0.82 | 1.72 | 0.365 |  | 1.14 | 0.78 | 1.65 | 0.498 |
| **CD8 T_EM_ (CD3^+^CD8^+^CD45^RO+^CD27^-^)** |  |  |  | 0.076 |  |  |  |  | 0.261 |
| Q2 & Q3 Combined (Reference) | 1.00 |  |  |  |  | 1.00 |  |  |  |
| Lower Quartile | 0.80 | 0.55 | 1.17 | 0.249 |  | 0.91 | 0.61 | 1.34 | 0.616 |
| Upper Quartile | 1.29 | 0.92 | 1.82 | 0.140 |  | 1.26 | 0.90 | 1.78 | 0.180 |
| **CD8 T_EMRA_ (CD3^+^CD8^+^CD45^RO-^CD27^-^)** |  |  |  | 0.190 |  |  |  |  | 0.651 |
| Q2 & Q3 Combined (Reference) | 1.00 |  |  |  |  | 1.00 |  |  |  |
| Lower Quartile | 0.72 | 0.50 | 1.03 | 0.069 |  | 0.84 | 0.57 | 1.24 | 0.376 |
| Upper Quartile | 0.93 | 0.65 | 1.34 | 0.700 |  | 0.91 | 0.64 | 1.31 | 0.622 |
| **CD8CD27-/CD8CD27+** |  |  |  | 0.029 |  |  |  |  | 0.235 |
| Q2 & Q3 Combined (Reference) | 1.00 |  |  |  |  | 1.00 |  |  |  |
| Lower Quartile | 0.60 | 0.41 | 0.88 | 0.010 |  | 0.69 | 0.45 | 1.06 | 0.091 |
| Upper Quartile | 0.98 | 0.69 | 1.39 | 0.906 |  | 0.97 | 0.68 | 1.38 | 0.861 |

Highlighted fields remain significant after correction for multiple testing

**
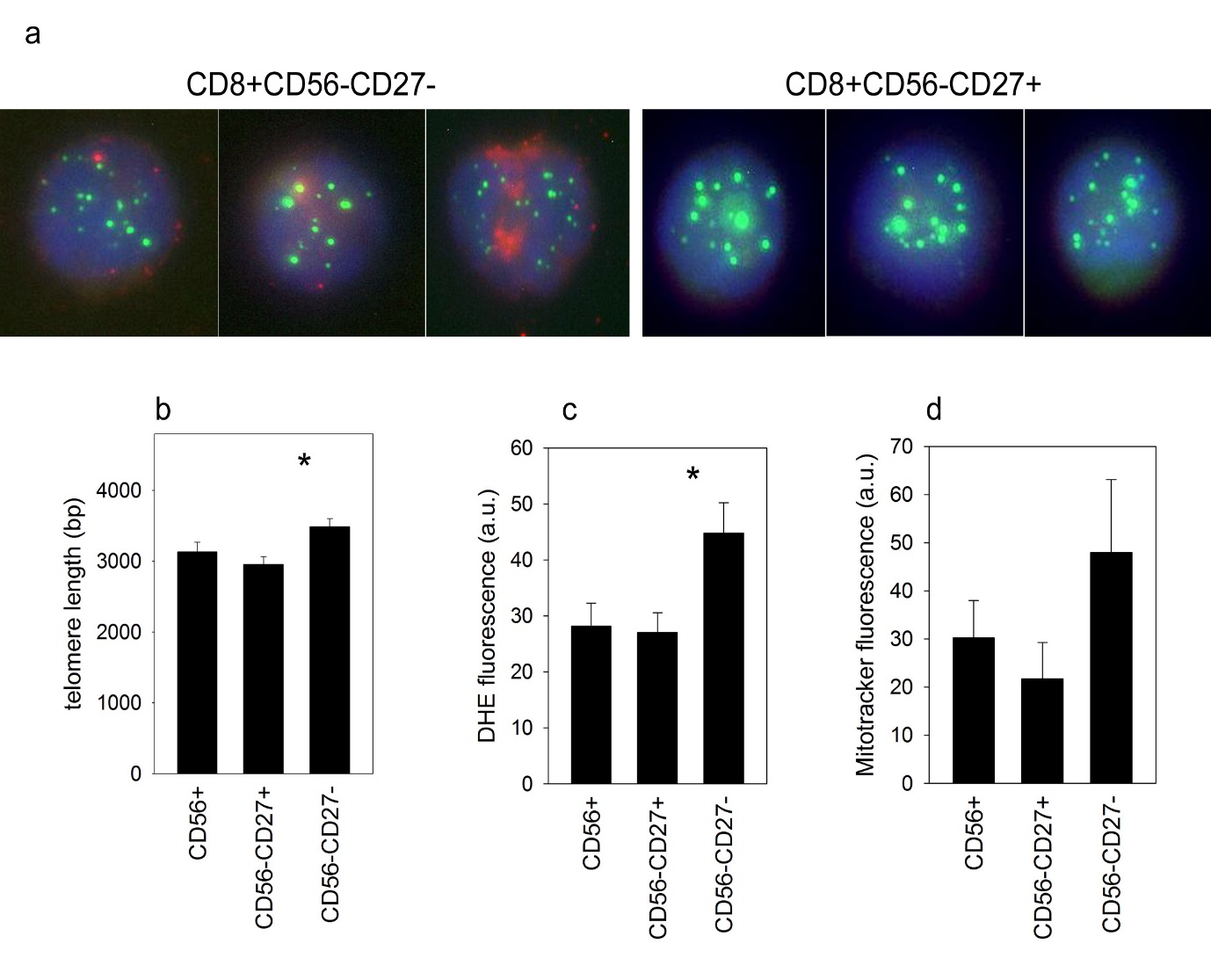
**

**Suppl. Fig. S1. A senescence-like phenotype in CD8+CD56-CD27- T cells. a) r**epresentative immunoFISH images (maximum intensity projections) of the indicated cell fractions. Green: telomeres, red: 53BP1 DNA damage foci, blue: DAPI. **b)** telomere length (qPCR), **c)** cellular ROS levels (DHE fluorescence intensity), **d)** mitochondrial mass (Mitotracker Green fluorescence intensity). The indicated fractions were magnetically sorted from CD8+ T cells from four volunteers, data are M ± SEM (n=4). Significant differences between CD27+ and CD27- CD8 T cells (ANOVA with post-hoc Holm-Sidak test, p<0.05) are indicated by *.

**
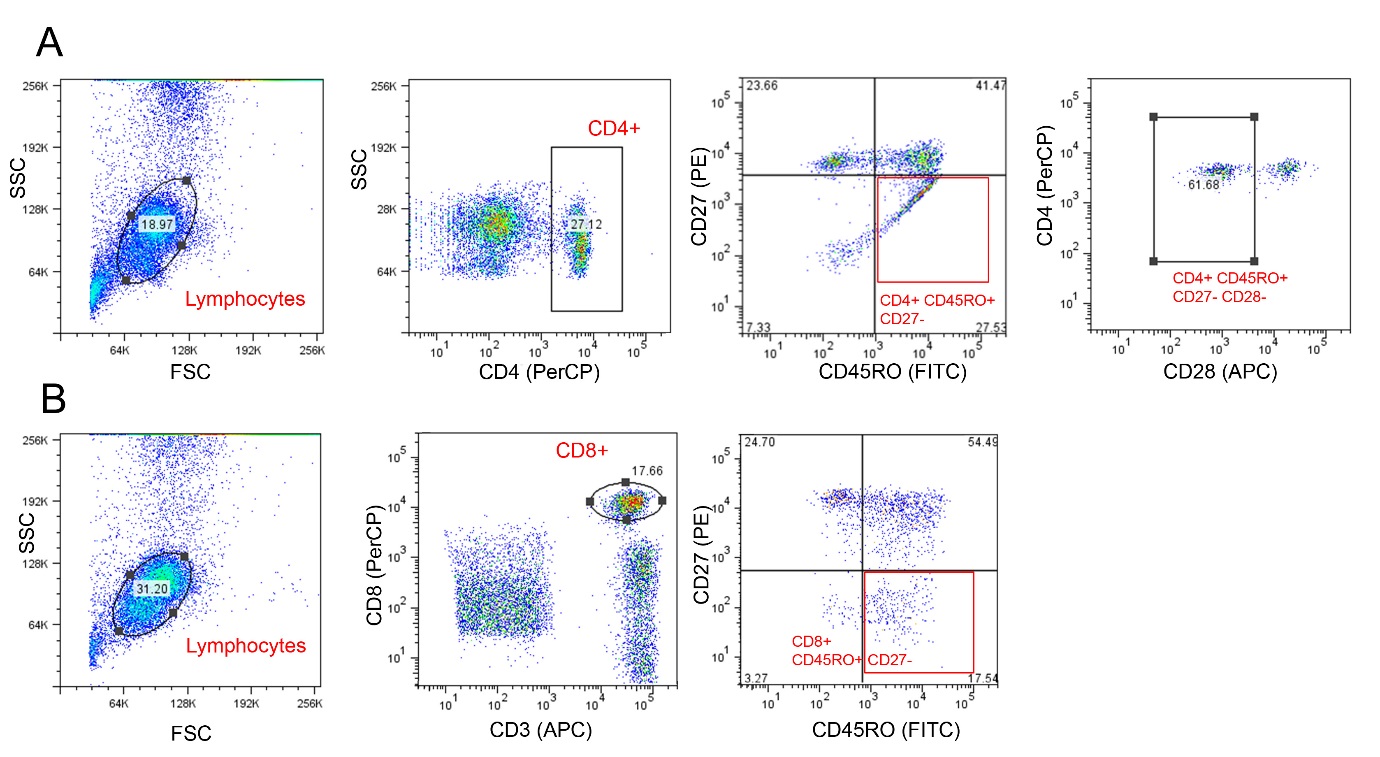
**

**Suppl. Fig. S2. Examples for the gating strategies for CD4 (A) and CD8 (B) T cells.** 2-dimensional flow cytometry histograms for the indicated marker combinations, gate positions and counts within the gates are shown.
